# Relevance or Excellence? Setting Research Priorities for Mental Health and Psychosocial Support in Humanitarian Settings

**DOI:** 10.3109/10673229.2012.649113

**Published:** 2012-02-15

**Authors:** Wietse A Tol, Vikram Patel, Mark Tomlinson, Florence Baingana, Ananda Galappatti, Derrick Silove, Egbert Sondorp, Mark van Ommeren, Michael G Wessells, Panter-Brick Catherine

**Affiliations:** 1 Global Health Initiative, and Sangath, Goa, India; 2 Jackson Institute for Global Affairs, and Sangath, Goa, India; 3 Department of Anthropology Yale University, and Sangath, Goa, India; 4 HealthNet TPO, Amsterdam, Netherlands; 5 London School of Hygiene & Tropical Medicine, and Sangath, Goa, India; 6 Stellenbosch University, South Africa; 7 School of Public Health, Makerere University, Uganda; 8 University of Colombo and Good Practice Group, Colombo, Sri Lanka; 9 School of Psychiatry, University of New South Wales, Australia; 10 Department of Global Health and Development, London School of Hygiene & Tropical Medicine, UK; 11 Department of Mental Health and Substance Abuse, World Health Organization, Geneva, Switzerland; 12 Mailman School of Public Health, Columbia University, USA

**Keywords:** disasters, global mental health, humanitarian crises, political violence, psychosocial support, research priorities

## Abstract

*Background:* Humanitarian crises are associated with an increase in mental disorders and psychological distress. Despite the emerging consensus on intervention strategies in humanitarian settings, the field of mental health and psychosocial support (MHPSS) in humanitarian settings lacks a consensus-based research agenda. *Methods:* From August 2009 to February 2010, we contacted policymakers, academic researchers, and humanitarian aid workers, and conducted nine semistructured focus group discussions with 114 participants in three locations (Peru, Uganda, and Nepal), in both the capitals and remote humanitarian settings. Local stakeholders representing a range of academic expertise (psychiatry, psychology, social work, child protection, and medical anthropology) and organizations (governments, universities, nongovernmental organizations, and UN agencies) were asked to identify priority questions for MHPSS research in humanitarian settings, and to discuss factors that hamper and facilitate research. *Results:* Thematic analyses of transcripts show that participants broadly agreed on prioritized research themes in the following order: (1) the prevalence and burden of mental health and psychosocial difficulties in humanitarian settings, (2) how MHPSS implementation can be improved, (3) evaluation of specific MHPSS interventions, (4) the determinants of mental health and psychological distress, and (5) improved research methods and processes. Rather than differences in research themes across countries, what emerged was a disconnect between different groups of stakeholders regarding research processes: the perceived lack of translation of research findings into actual policy and programs; misunderstanding of research methods by aid workers; different appreciation of the time needed to conduct research; and disputed universality of research constructs. *Conclusions:* To advance a collaborative research agenda, actors in this field need to bridge the perceived disconnect between the goals of “relevance” and “excellence.” Research needs to be more sensitive to questions and concerns arising from humanitarian interventions, and practitioners need to take research findings into account in designing interventions. (Harv Rev Psychiatry 2012;20:25–36.)

## BACKGROUND

In 2009 alone, more than 119 million people were affected by natural disasters.^1^ In that year, the world also experienced 36 active armed conflicts in 26 different countries.^2^ A number of systematic reviews and meta-analyses have documented the mental health and psychosocial status of communities residing in settings of humanitarian crisis, encompassing a broad range of emergencies, including natural disasters, industrial accidents, and complex emergencies related to political violence.^3^–^8^ Such reviews highlight mental health consequences that range from nonpathological psychological distress to mental disorders, including posttraumatic stress disorder, major depressive disorder, generalized anxiety disorder, substance use disorders, and adjustment disorder. They also bear witness to the resilience shown by a majority of individuals within affected populations—namely, the capacity to retain good mental health and psychosocial well-being despite exposure to significant adversity 9,10 In addition, authors have noted detrimental effects of humanitarian crises on social contexts.^11^

Four years ago, widely endorsed consensus guidelines were published to guide the implementation of mental health and psychosocial support (MHPSS) in humanitarian settings (the term *settings* is used here to refer to the locations in which humanitarian crises occur).^12^ Researchers remain divided on a number of issues, however, including: (1) the distinction between mental disorder and non-disordered psychological distress,^13^,^14^ (2) which stressors matter most for poor mental health (those specifically engendered by humanitarian crises vs. those engendered by structural contexts [in particular, prevailing social, economic, and political stressors]),^15^,^16^ and (3) the importance and cultural validity of the PTSD diagnostic category.^17^ In addition, mental health work in humanitarian settings remains plagued by a small evidence base for prevention and treatment options.^18^

Given both ideological and knowledge gaps in the existing literature, empirical research is needed to guide these debates with greater evidence. Establishing a consensus-based research agenda would make a specific contribution to conducting such research activities in a more coordinated, coherent, and cost-effective manner. To this end, the Mental Health and Psychosocial Support in Humanitarian Crises—Research Priority Setting (MH-SET) project was initiated in 2008 using a consensus-building methodology previously implemented by the Child Health and Nutrition Research Initiative (CHNRI).^19^ This consensus-building methodology provides an opportunity for a representative group of experts to formulate research priorities. This structured process involves (1) the systematic listing of research questions and (2) ranking of research questions using predetermined criteria (e.g., whether answering research questions would lead to improved practice, or whether the questions can be answered in an ethical manner).

In addition to implementing the CHNRI methodology, the steering group of MH-SET was especially interested in including voices of those with experience and expertise in locations that are characteristically remote from the main seat of humanitarian decision making (i.e., those *not* in high-income countries or the capitals of countries where humanitarian decisions are often made). Although calls for “local ownership” and “participation” are increasing in humanitarian practice,^20^ numerous challenges remain if these principles are to be translated into practice. Especially with regard to research activities in humanitarian settings, little effort has been made to increase the participation of researchers in low- and middle-income countries (LAMICs), despite concerns about power differences between international, national, and local humanitarian stakeholders.^21^

This article describes a study aiming to document local experts' perspectives on mental health and psychosocial research priorities in humanitarian settings. We aimed to (1) investigate research priorities for mental health interventions and psychosocial support from the perspective of practitioners and researchers in LAMICs affected by humanitarian crises, (2) compare the perspectives of respondents who work in remote humanitarian settings and those who hold health-related policy and academic positions within LAMICs, and (3) assess perspectives on the factors that facilitate or hamper research efforts in humanitarian settings.

## METHODS

### Study Design

The study was directed by a ten-member steering group of the MH-SET initiative—namely, the present authors. We purposively selected three LAMICs, from Asia, Latin America, and Africa, that were recently affected by humanitarian crises. The MH-SET steering group invited three national experts to undertake the role of in-country team leaders to implement this research. The national experts were recruited through the professional networks of MH-SET on the basis of the following criteria: (1) experience in conducting research with relevance to MHPSS in humanitarian settings, (2) a central role in organizing such research (indicated by their positions in well-established organizations and relational networks), and (3) their ability to organize quality focus groups (indicated by previous success in conducting focus groups). Team leaders contributed to the logistical and thematic planning of the focus groups, including the location, participants, and format. They sent invitations to particular individuals and organizations that played important roles as stakeholders in MHPSS research and service provision in humanitarian settings within the country under consideration. None of the contacted individuals or organizations refused participation.

In each country, focus groups were held (1) in the capital, with policymakers and academics in health- and other services-related work, and (2) in remote districts where humanitarian services were currently being implemented, with humanitarian aid workers at their work sites. All discussions lasted two to three hours and were taped and transcribed (Spanish and Nepali text was transcribed in English; in Uganda, discussions were conducted in English). Research procedures were in accordance with the Helsinki Declaration. All participants were briefed regarding the purpose of the study, were guaranteed anonymity, and signed informed consent. The study received formal ethical approval from Durham University and received in-country support from national institutions.

### Sites and Participants

Over the period August 2009 to February 2010, the research team contacted in-country team leaders, issued invitations to potential participants, translated materials, conducted focus groups (Peru, August 2009; Uganda, November 2009; Nepal, February 2010), and completed thematic analyses. An overview of participant characteristics is provided in Table 1.

**Table 1:** Structure and Participants in Nine Focus Groups

	Capitals	Humanitarian settings
Peru	P_cap_:	P_hum:_
	*n* = 13 (4M/9F)	*n* = 9 (4M/5F)
	Predominantly psychologists (10), but also a lawyer, teacher, and policymaker	All participants were trained as psychologists, and some worked additionally as lawyers, teachers, and managers with the UN or INGOs
	Participants worked with the government (3), INGOs (4), universities (2), and the UN (1), some in combination (3)	Participants worked with the government (3), INGOs (3), universities (2), and the UN (1)
Uganda	U_cap_:	U_hum1_:
	*n* = 10 (4M/6F)	*n* = 10 (7M/3F)
	Group divided between participants with senior positions in government health care (4), academic affiliations (3; psychiatry, psychology, medical anthropology) and senior/managerial positions in NGOs (3)	Majority of participants working with NGOs (6)
		Others (4) employed by the government (psychiatrist, clinical officer, and nurse) or Gulu University
		U_hum2_:
		*n* = 12 (9M/3F)
		Majority of participants working with NGOs in diverse positions (7; child protection, education, mental health)
		Others (5) working as psychiatric clinical officers within government health centers
Nepal	N_cap1_:	N_hum1_:
	*n* = 17 (8M/9F)	*n* = 16
	Large group of participants associated with NGOs/INGOs (10)	All participants working with organizations involved in assisting populations affected by the Koshi floods, either with NGOs (12) or the government (4)
	Others affiliated with universities (4) or the government (3)	
	N_cap2_:	N_hum2_:
	*n* = 14 (8M/6F)	*n* = 13 (7M/6F)
	All researchers working at the national level, with all but one affiliated with NGOs/INGOs	All psychosocial care providers working with the designated agency active in providing psychosocial care in the Bhutanese refugee camps

F, female; INGO, international nongovernmental organization; M, male; NGO, nongovernmental organization; UN, United Nations.

In Peru, we conducted two focus groups, one in the capital Lima (P_cap_) and the other in Chinchha (P_hum_), in cooperation with an independent consultant who played a central role in the humanitarian response to the earthquake that affected Peru on August 15, 2007. That earthquake (with the epicenter in southern Peru and a magnitude of 7.9 on the Richter scale) and the associated tsunami resulted in 593 deaths and directly affected over 600,000 other persons.^22^

In Uganda, we conducted one focus group in the capital Kampala (U_cap_) and two in northern Uganda (Gulu [U_hum1_] and Lira [U_hum2_]). These encounters were facilitated by the Transcultural Psychosocial Organization Uganda (a national nongovernmental organization providing MHPSS for vulnerable populations across Uganda). Northern Uganda suffered an armed conflict between the Lord's Resistance Army and the government's Uganda People's Defense Forces from 1987 until 2006. Thousands were killed in the conflict, which, at its peak in 2005, displaced 1.8 million people.^23^

In Nepal, we conducted two focus groups in the capital Kathmandu and two in humanitarian areas. In these settings, the humanitarian crisis was related to a variety of causes, including floods, an influx of refugees from Bhutan in the 1990s, and a Maoist armed insurgency between 1996 and 2006. We worked in coordination with the National Mental Health Network, a collaborative network consisting of nongovernmental organizations and government agencies aimed at strengthening mental health and psychosocial care across Nepal. In Kathmandu, one focus group comprised representatives from the organizations involved in the National Mental Health Network (N_cap1_), and the other comprised academic researchers working within national institutions (N_cap2_). The third focus group was convened in Sunsari (N_hum1_), a district in southern Nepal. On August 18, 2008, the Koshi river—a large, transboundary river flowing in Nepal and India—broke its embankments in southern Nepal, flooded several districts, and affected over 42,000 Nepali citizens. This area was already affected by a Maoist insurgency from 1996 to 2006, with loss of life exceeding 16,000.^24^ The fourth focus group was convened with humanitarian aid workers in Bhutanese refugee camps (N_hum2_). Approximately 100,000 Nepali-speaking Bhutanese refugees fled Bhutan from the early 1990s and have since been living in UN-administered camps in southeastern Nepal.^25^

### Data Collection

We conducted focus groups rather than individual interviews in order to facilitate debate and assess whether a consensus could be achieved among groups of participants. Using a semistructured format, our group discussions had three general themes: (1) to generate and discuss the ten research questions that would most strongly contribute to MHPSS in humanitarian settings, (2) to obtain the participants' perspectives on conducting research in settings where MHPSS is implemented, and (3) to identify the perceived obstacles and facilitating factors in conducting research in such settings.

### Analyses

The data were entered in English into NVivo 9. Thematic analysis was then undertaken on the text based on grounded theory; that is, in developing a “theory” about how MHPSS research is prioritized in humanitarian settings, we started from the focus groups' participants' expressed viewpoints in the raw data.^26^ After reading all materials, two analysts working independently produced a coding scheme based on the main themes emerging from the text. That scheme focused on inductive categorizations of (1) the research priorities generated by participants, (2) the prose text in which research priorities were discussed and prioritized, and (3) the narratives related to factors that may impede or facilitate research. Findings were contrasted over the different sites, which we have designated with the subscripts cap (for capitals) and _hum_ (in sites where humanitarian services were implemented) after the first letter designating the countries in question (i.e., N, P, U). Applying a deductive approach, we then coded and checked the data, further refining themes and subthemes. Comparison of thematic coding schemes across analysts revealed a limited number of differences, which were resolved to form a final list of codes. In-country team leaders reviewed and commented on the write-up of all final analyses.

## RESULTS

### Prioritized Research Questions

In each focus group, participants agreed upon a list of the ten most pressing research concerns to strengthen MHPSS in humanitarian settings. These concerns fell into five research themes (see Table 2).

**Table 2:** Overview of Prioritized MHPSS Research Themes in Humanitarian Settings

Research theme	Focus group	Priority rankings (1–10)	Examples of research questions proposed by participants
Assessing the prevalence and burden associated with mental health and psychosocial problems	P_cap_	6	“How can psychiatric diagnostic procedures for people affected by disasters be improved?”
	P_hum_	7	“What kind of psychosocial problems emerge or worsen after a disaster? How do they affect different subgroups of the population?”
	U_cap_	2, 4, 5, 6	“What is the biopsychosocial impact of climatic change?”
			“What are the mental health challenges associated with epidemics and emerging diseases?”
	U_hum1_	1, 6, 8, 9	“What are the long-term mental health and psychosocial effects of children being exposed to armed conflict?”
			“What is the burden of mental illness in crisis-affected populations, and what are the costs associated with that burden?”
	U_hum2_	7, 10	“What is the relationship between livelihood and mental health/psychosocial well-being?”
	N_cap1_	1	“What are the prevalences of mental disorders in humanitarian settings?”
	N_cap2_	1, 2, 3	“Which population groups have suffered the worst mental health and psychosocial impact due to the armed conflict?”
			“What are the specific mental health and psychosocial problems of internally displaced people?”
	N_hum1_	1, 4, 5, 6	“What are the most common mental health and psychosocial problems, along with their causes, in humanitarian settings?”
			“How many people have psychosocial problems in humanitarian settings?”
Improving MHPSS implementation	P_cap_	1, 2, 3, 7, 9, 10	“How can community participation in interventions best be secured?”
			“How can emotional support for disaster-response teams be put in place during and after interventions?”
	P_hum_	1, 5, 6, 8, 10	“What mechanisms contribute to optimal coordination between institutions (public and private) involved in emergency response and reconstruction?”
			“What mechanisms help to ensure sustainability of all activities done by all institutions (NGOs and aid workers) and to transfer them successfully to the relevant health authorities?”
	U_cap_	7, 8, 9	“What is the role of traditional systems in mental health care, justice, conflict resolution, and psychosocial support?”
			“How can health systems be strengthened in an integrated fashion for provision of mental health care and psychosocial support?”
	U_hum1_	2, 3, 7	“How do we care for the carers (including mental health workers as well as families and communities caring for those with mental illnesses)?”
			“To what extent are traditional healers (both herbalists and spiritual healers) involved in treating mental health and psychosocial problems?”
	N_cap1_	2, 4, 6, 7, 9, 10	“What are the currently existing services for mental health?”
			“What role is played by civil society in addressing mental health and psychosocial issues in humanitarian settings?”
	N_hum1_	2, 3, 8	“What are people's expectations and interests with regard to mental health and psychosocial support in humanitarian settings?”
			“Which organizations are currently providing mental health and psychosocial services?”
	N_hum2_	1, 2, 3, 5, 6, 7, 10	“Which generic tools and techniques may be used by service providers in humanitarian settings to improve mental health and psychosocial well-being?”
			“What are appropriate methods to raise awareness on mental health and psychosocial issues in communities affected by disasters?”
Evaluating MHPSS interventions	P_cap_	4, 5	“To what extent and through which mechanisms are interventions effective?”
			“What are participants' views on the effectiveness of interventions?”
	P_hum_	4, 9	“How are parents and the social environment influenced by psychosocial activities conducted with children?”
			“How can we highlight the results that occur through interventions in the community?”
	U_cap_	1, 3, 10	“What intervention models of care are effective in postconflict situations in Uganda?”
			“What models of care (traditional and modern) are effective in rehabilitation and resettlement of orphans and vulnerable children?”
	U_hum1_	5	“What are the most effective interventions for treating and preventing mental disorders and psychosocial distress?”
			“How can we effectively monitor and evaluate the various types of interventions?”
	U_hum2_	1, 8, 9	“Which interventions actually contribute to psychosocial well-being?”
			“What is the relative efficacy of traditional treatments and modern treatments in humanitarian settings?”
	N_hum1_	7, 9, 10	“What are the most effective awareness-raising programs?”
			“What are people's perceptions regarding mental health and psychosocial support after receiving services?”
Risk and protective factors	U_hum1_	4, 10	“How does substance abuse contribute to mental illness?”
			“What is the relationship between general humanitarian aid and mental health/psychosocial well-being?”
	U_hum2_	2, 3, 4, 6	“What is the relationship between armed conflict and breakdown in cultural norms?”
			“What are the main causes for the high level of neurological problems seen in northern Uganda?”
	N_cap1_	3, 5, 8	“What are relevant cultural practices, and how do they affect mental health and psychosocial well-being in humanitarian settings?”
			“How do issues of reconciliation, recovery, and reintegration affect mental health and psychosocial well-being in humanitarian settings?”
	N_cap2_	4, 5, 6, 7, 8, 9, 10	“What is the impact of domestic violence on mental health and psychosocial well-being in humanitarian settings?”
			“What are the coping strategies used by people in humanitarian settings, and how do they affect mental health and psychosocial well-being?”
	N_hum2_	4, 8, 9	“How does gender affect mental health and psychosocial well-being in humanitarian settings?”
			“How does stigma affect mental health and psychosocial well-being in humanitarian settings?”
Improving research methods and processes	P_cap_	8	“What are appropriate methods for conducting research on disaster-affected populations? For example, how could quantitative research be supplemented with qualitative data?”
	P_hum_	2, 3	“What qualitative result indicators can be used to highlight the successes of our postdisaster intervention projects?”
			“How can we measure the positive impact of play areas and care areas offered to children in emergency situations?”
	U_hum2_	5	“What are appropriate indicators for psychosocial well-being in both adults and children?”

MHPPS, mental health and psychosocial support; NGO, nongovernmental organization.

Overall, the participants placed a strong emphasis on research questions related to *assessing the prevalence and burden associated with mental health and psychosocial problems* (e.g., prevalence of mental disorders, common psychosocial problems, and the wider impact of mental health problems on productivity, livelihoods, family and community functioning). This research theme was prioritized in all focus groups except one (N_hum2_), and especially in Uganda and Nepal, both by participants in capitals (e.g., N_cap2_) and in areas where humanitarian services were being implemented (e.g., U_hum1_; N_hum1_). One participant discussed increased knowledge on this theme as a prerequisite to interventions, as follows:

We depend on data published by the WHO and World Bank on low- and middle-income countries. But if we talk specifically about Nepal, we don't know what number of people are mentally ill or have mental problems. Until we know that, we don't know what is needed, how much is needed, how to do this, etc. [N_cap1_]

A second important theme concerned *research on how implementation of MHPSS may be improved* (e.g., research questions related to community participation and building on existing supports, strengthening coordination, sustainability, selection and training of human resources, policy frameworks); this was the most important research theme in 4 out of 9 focus groups (P_cap_, P_hum_, N_cap1_, N_hum2_), and featured in all but two focus groups (U_hum2_, N_cap2_). In Peru particularly, participants emphasized the importance of research that enabled greater understanding of, and engagement with, emic perspectives and community concerns, in order to inform more participative and community-appropriate interventions. For example, one participant stated:

I believe intervention and research should involve affected people in order to know firsthand what they are thinking, what they are feeling, in order to know the main trends. [P_cap_]

This theme was also discussed in detail during the Ugandan focus groups, particularly in relation to the importance of traditional healing.

Research would help us a lot, actually, to understand our communities. You realize that communities are very active in mental health and psychosocial interventions … They have their own programs, so it is important for us to understand the traditional interventions so that we can develop or come up with a cultural approach. [U_hum2_]

In addition, participants were particularly interested in research that could help improve aspects of service delivery in which they observed specific problems, such as the coordination between agencies, sustainability of services and supports, and need for research to document best practices for future. For example:

Even now, none of the municipalities is working on a contingency plan. It is very regrettable that, in spite of the experiences we went through, we didn't seize the opportunity to get something good out of this. [P_hum_]

A third widely occurring theme referred to the *evaluation of MHPSS interventions*. The main question here concerned the identification of those supports that most improve well-being (e.g., the efficacy and effectiveness of interventions, intervention mechanisms, and the wider impact of interventions on families and communities). This theme was prioritized in all focus groups in Peru (*n* = 2) and Uganda (*n* = 3), and in one out of the four focus groups in Nepal (N_hum1_). In their discussion on this theme, participants often focused on their experience of the somewhat haphazard selection of intervention modalities during humanitarian crises, without this selection being based on empirical evidence.

My experience here is that most people are just gambling. Development workers come with interventions without doing research. As such, their work does not actually impact people so much. [U_hum2_]

A fourth theme pertained to *risk and protective factors for mental health and psychosocial well-being*. Here, research on risk and protective factors concerned both individual-level factors (e.g., gender, age, coping behaviors) and factors in the wider social-ecological context (e.g., a breakdown in cultural norms, or issues of reconciliation and reintegration of former fighters in postconflict contexts in Nepal and Uganda). Research on risk and protective factors was prioritized in five focus groups (U_hum1_, U_hum2_, N_cap1_, N_cap2_, N_hum2_). Participants in some of the focus groups were especially interested in how, in their interventions, they may build on existing social support networks. Interest in these factors was often expressed with a view to addressing the causal chain of mental health problems.

I propose there should be research to help us establish the cause of mental health problems in Uganda. And once we have established the cause we will be able to address the root causes of mental health problems. [U_hum2_]

Finally, a fifth theme addressed *improving research methods and processes*, such as improvements in the measurement of mental health and psychosocial well-being (e.g., psychiatric diagnostic procedures and appropriate indicators of well-being), as well as questions on how to improve MHPSS evaluation. Improvements to research methods and processes were discussed in three focus groups (P_hum_, P_cap_, U_hum2_). For example:

I am interested to see how we can develop indicators of psychosocial well-being … so that we can use them as a basis upon which interventions are measured. If you are saying it is school enrollment, then that becomes your basis. If you are saying improved social functioning, whatever else you are doing is at least measured, and those indicators should be developed ecologically or through a process of collecting evidence and looking at how communities would put this. [U_hum2_]

### Perceived Obstacles

Participants in the focus groups presented a number of obstacles that, they felt, impede research that could help guide MHPSS in humanitarian settings. First, participants spoke of a “disconnect” between the priorities of those at the central level (in capitals; i.e., policymakers and researchers) and those at the implementation sites (i.e., aid workers). The latter expressed the feeling that policymakers and academics were not well attuned to the actual needs on the ground, and that research and interventions were therefore not focused on the main problems of those affected by humanitarian crises.

The central level does research and goes away. They don't acknowledge field-level people. [N_hum2_]

This concern was sometimes shared by the policymakers and academicians.

In the context of Nepal, the grassroots-level workers in mental health and psychosocial issues are the community psychosocial workers and the counselors. If their experiences are included, then things may change, but from the policy level no change comes. [N_cap1_]

This concern was often linked to a worry about the unsustainability of programs that had been initiated at the central level without consulting the people working in actual humanitarian sites.

**Figure 1:**
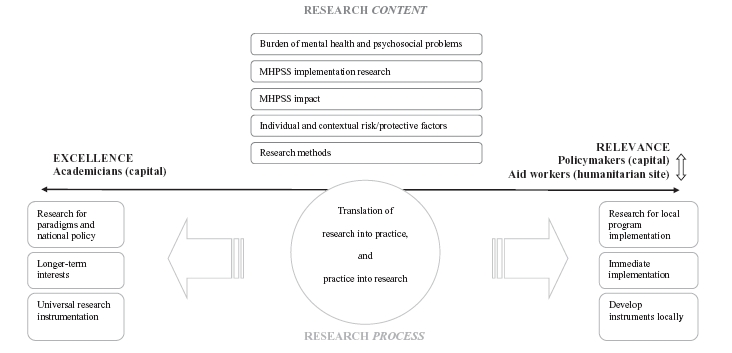
Research priorities: agreement on context, disagreement on process.

Second, we observed a disconnect between academicians, on the one hand, and policymakers and aid workers, on the other. We interpreted this disconnect in terms of “relevance” and “excellence” (Figure 1).

On this interpretation, policymakers and aid workers emphasize the utilitarian value of research—that is, its capacity to be transformed into immediate solutions (= relevance), whereas academic researchers give more weight to the scientific validity of the method and results (= excellence). Conflicts in balancing relevance and excellence may arise because of differences between researchers and practitioners regarding priorities (which problems are of greatest concern?), time management (time pressure to implement programs vs. time needed to consider all aspects of the topic), language and communication (easily comprehensible descriptions in an accessible forum vs. precise language expressed in the format of a specialized discipline), integration (comprehensive answers to complex issues vs. well-founded answers to specific questions), and the final research product (translation into policy and programs vs. publication in peer-reviewed journals).^27^ For example, academicians in this study were concerned about the lack of understanding of research among aid workers, and disagreed with aid workers on the time frame of research. Researchers were worried that aid workers were too much concerned with the short-term outcomes of research without taking a longer-term view.

It is because researchers work for the long term. They think there can be a solution for the long term if they find out the reasons. Grassroots-level workers do not care for the future; they want something immediately. [N_cap1_]

This sentiment was echoed by the practitioners themselves—for instance, in Gulu, Uganda:

We—the practitioners on the ground—we see research as something that is time wasting; it takes long. But we are comfortable with assessments that give immediate results. [U_hum1_]

Furthermore, concerns were raised regarding the appropriateness and impact of research conducted by outsiders— particularly by researchers associated with universities in high-income settings. Some participants expressed frustration that research projects were being optimally geared not toward informing programs but rather toward informing academic debates without relevance for practice.

First of all, these academicians have mostly theoretical knowledge. Tactically, those who are in the field doing the real work understand the situation much better. [U_hum1_]

There were specific concerns, shared in both capitals and humanitarian settings, that research results were not being disseminated or being translated into improving the programs.

I believe research is meant to offer some sort of support to the generation of a culture of mental health prevention. In terms of research, I do not believe that there is a dissemination of the little we have done. [P_cap_]

When this research is conducted, there is no feedback. It's never brought back to the community, and the community keeps on demanding to know the results of the research. [U_hum2_]

Also, concerns were raised about the appropriateness of tools from Western settings in non-Western settings. Whereas researchers emphasized tools that may contribute to a comparison across cultural settings, practitioners (especially aid workers) doubted whether such tools generated locally valid results.

Normally, the concept is in English. When you go down to the common man, you have to ask in the local language. In the process of translation, the content is watered down so you may end up not getting what you would have got if you had asked the question in English. [U_hum1_]

We summarize our findings in Figure 1. In the upper half of the figure, we state research priorities in terms of content. In the lower half, we state characteristics of research processes in terms of contrasts established by the participants between excellence and relevance. We found consensus with respect to priority research questions, despite some variation across countries with regard to questions of interest. Thus, similar research themes were prioritized by policymakers, academicians, and aid workers both in capitals and in remote settings. Differences arose, however, with regard to how, and with what aims, research should be conducted. Researchers were more interested in the quality or excellence of the research, whereas practitioners (policymakers and aid workers) were more concerned that the research be useful on the ground.

## DISCUSSION

We documented perspectives on research priorities with policymakers, academics, and aid workers engaged with humanitarian work or crisis relief work in three LAMICs. A principal finding is that, with some exceptions, similar research themes were prioritized across countries and between participants in capitals and humanitarian settings. In order of frequency, these themes pertained to (1) the prevalence and burden associated with mental health and psychosocial problems, (2) implementation (e.g., research on human resources, sustainability, coordination, existing resources), (3) evaluation of MHPSS programs, (4) protective and risk factors for mental illness, and (5) improved research methods and processes. Researchers and practitioners disagreed on aspects of how research should be conducted.

With regard to the stated research priorities, there are similarities with other health fields. For instance, the expressed interest in implementation research resonates with comparable calls in maternal and child health.^28^ Likewise, researchers have previously advocated the importance of identifying the social determinants of health.^29^,^30^ The emphasis on research that addresses the burden of mental health and psychosocial problems in humanitarian crises might be considered surprising, given the larger body of research that has been published on this issue (for meta-analyses, see Steel et al. [2009]^3^ and Attanayake et al. [2009]^6^) and the contention that has surrounded the importance of psychiatric epidemiological research for informing MHPSS response.^12^,^31^

With regard to the identified disconnect concerning the relevance versus excellence of research, our findings accord with the observed tension between these two factors as judged by different sectors involved in humanitarian work.^27^ Researchers valued the scientific validity of findings, employed a longer time frame, and valued comparability of findings across settings, whereas practitioners valued research if it could benefit local and immediate program implementation. As our interest was mainly in how MHPSS research was prioritized, we highlighted this specific disconnect in Figure 1. It should also be remembered, however, that a gap was observed between the practitioners themselves—that is, between policymakers at the central level and aid workers in remote humanitarian settings.

The study findings must be interpreted in light of a number of limitations. First, we identified in-country team leaders through the network of the MH-SET steering group: this method may have led to the selecting of team leaders who favored current research initiatives in humanitarian settings, leading to an underrepresentation of other perspectives. Second, since our sample size was small, generalizability of findings within Peru, Nepal, and Uganda and to other LAMIC settings is unknown. Even so, in view of the consistent findings about the priority of research themes across different settings, that particular finding would appear to be reliable. Third, due to time constraints we did not consult the recipients of humanitarian services regarding their research priorities.

A strength of this study is its targeted effort to integrate the perspectives of an interdisciplinary range of national and local voices on research—in particular, by conducting focus groups in various settings in three countries. Although *local ownership* is a common catch phrase in humanitarian work, actual implementation of this imperative has been slow.^32^ Research on local perceptions of humanitarian aid is emerging, but it is still unsystematic.^33^ We know of no studies focused on MHPSS research. This study therefore takes a first step toward advancing the principle of building local ownership of research in relation to mental health in humanitarian settings—a need specifically identified by participants in this study—by investigating the perspectives of local stakeholders working in academic, policy, and implementation settings.

An important and challenging question arising from our findings is how the identified disconnect between relevance and excellence may be bridged to ensure that future MHPSS research is both practically relevant *and* scientifically excellent. This concern, which has resurfaced in the literature at various times, is currently a salient issue in the context of research on evidence-based decision making.^34^ This problem is obviously not limited to MHPSS in humanitarian settings. Given the acute needs for service delivery in humanitarian crises, however, the stakes for achieving this balance for MHPSS research in such settings are arguably higher than in other settings.

The literature suggests a number of ways forward. First, increased communication and collaboration between researchers and practitioners is obviously important.^35^ Efforts at stimulating collaboration, though, should be mindful of power differences between partners in research projects and, in order to achieve sustained capacity building, should preferably be undertaken over the long term.^28^ Second, the majority of research on bridging the gap between evidence and practice appears to have focused on the uptake of clinical guidelines by health professionals. For example, one systematic review of this literature identified the effectiveness of audit and feedback strategies, as well as education and training efforts.^36^ Similarly, a recent systematic review found that the strongest determinant for uptake of research by nurses was their attitudes toward research.^37^ Although the uptake of research-based clinical guidelines by those working on the ground is imperative (i.e., translating research into practice), it will be just as important to ensure that academic researchers are guided to answer questions that are deemed the most significant by practitioners. One way to achieve this result may be the increased engagement in research activities of those who are implementing services—for example, through systematic uptake of research by implementing agencies while providing services.^38^ As Walley and colleagues^39^ note, it is a matter of getting practice into research. Such an effort will require sustained infrastructure strengthening, research training, and, given the current lack of human resources for global mental health, capacity building in both practice and research fields.^40^ Finally, bridging gaps between the goals of research excellence and research relevance will require listening to “local voices”—for example, the communities affected by humanitarian crises.

In conclusion, this study explored national and local perspectives on research priorities for MHPSS in humanitarian settings. Findings indicate that sustained efforts to foster research that demonstrates both excellence and relevance are necessary to bridge the disconnect between practitioners and researchers—for example, by bringing research into practice and by engaging practitioners in research. The finding that similar research themes were prioritized by these different stakeholder groups provides an indication that researchers and practitioners share sufficient common ground to establish a collaborative research agenda. For that to happen, however, funding structures are needed to strengthen the infrastructure for translational research. Answering highly prioritized research questions is not enough.

We gratefully acknowledge Miryam Rivera Holquin, Patrick Onyango, Emmanuel Ngabirano, Nawaraj Upadhaya, and Nagendra P. Luitel for facilitating data collection, and Tim P. Williams for his assistance with data analysis.
